# Sweet-spot operation of a germanium hole spin qubit with highly anisotropic noise sensitivity

**DOI:** 10.1038/s41563-024-01857-5

**Published:** 2024-05-17

**Authors:** N. W. Hendrickx, L. Massai, M. Mergenthaler, F. J. Schupp, S. Paredes, S. W. Bedell, G. Salis, A. Fuhrer

**Affiliations:** 1 IBM Research Europe - Zurich, Rüschlikon, Switzerland; 2 IBM Quantum, T.J. Watson Research Center, Yorktown Heights, NY USA

**Keywords:** Quantum information, Qubits, Electronic devices, Electronic properties and materials, Quantum dots

## Abstract

Spin qubits defined by valence band hole states are attractive for quantum information processing due to their inherent coupling to electric fields, enabling fast and scalable qubit control. Heavy holes in germanium are particularly promising, with recent demonstrations of fast and high-fidelity qubit operations. However, the mechanisms and anisotropies that underlie qubit driving and decoherence remain mostly unclear. Here we report the highly anisotropic heavy-hole *g*-tensor and its dependence on electric fields, revealing how qubit driving and decoherence originate from electric modulations of the *g*-tensor. Furthermore, we confirm the predicted Ising-type hyperfine interaction and show that qubit coherence is ultimately limited by 1/*f* charge noise, where *f* is the frequency. Finally, operating the qubit at low magnetic field, we measure a dephasing time of ${T}_{2}^{* }$ = 17.6 μs, maintaining single-qubit gate fidelities well above 99% even at elevated temperatures of *T* > 1 K. This understanding of qubit driving and decoherence mechanisms is key towards realizing scalable and highly coherent hole qubit arrays.

## Abstract (web summary)

The authors report the sweet-spot operation of germanium hole spin qubits, exploring the optimization of the external magnetic field orientation, the *g*-tensor and its electric tunability, and hyperfine interactions.

## Main

The development of a fault-tolerant quantum computer^1^ that is able to solve relevant problems^2^ requires the integration of many highly coherent qubits. Spin qubits based on quantum dots^3^ hold excellent promise for scaling towards large-scale quantum processors, due to their small footprint and long coherence. In particular, hole qubits in strained germanium quantum wells have gained a strong interest over recent years^4^, with demonstrations of single-^5–7^ and multi-qubit^8,9^ operations as well as first steps towards operating large, multiplexed qubit registers^10^. This surge of interest is rooted in the combination of favourable properties that are possessed by holes in germanium: a low-effective mass that eases device fabrication^11^, a low-noise qubit environment^12^ and excellent quantum-dot control^13^, without the complication of low-energy valley states that have hindered progress for electrons in silicon.

The spin properties of valence band holes can be highly anisotropic^14–18^, resulting in a field-dependent coupling to the two dominant sources of decoherence in spin qubits: nuclear spin fluctuations^19^ and charge noise^20^. These anisotropies present both opportunities and challenges for building a scalable qubit platform. For example, the anisotropic heavy-hole *g*-tensor can amplify small variations in quantum-dot confinement, leading to site-dependent qubit properties^8,17^ and increasing requirements on material uniformity. However, when well-controlled, the anisotropy enables operational sweet spots where qubit control is maximized while decoherence is minimized^15,21–23^, overcoming the general trade-off between qubit control and coherence. Theoretical considerations predict the operating point of such sweet spots to depend on specific material and device parameters such as strain^24^ or geometry^25^, although an experimental demonstration of the heavy-hole anisotropies and their implications on qubit performance is lacking.

Here we unveil the mechanisms that enable qubit driving and mediate decoherence in germanium hole qubits. We fully characterize the heavy-hole *g*-tensors of a two-qubit system and their sensitivity to electric fields. A comparison with the dependence of qubit coherence and Rabi frequency on the orientation and magnitude of the external magnetic field demonstrates that both qubit driving and charge-noise-induced qubit decoherence are explained by the distortion of the *g*-tensor through electric fields. Furthermore, we confirm the predicted Ising character of the hyperfine interaction between the heavy-hole spin and the ^73^Ge nuclear spin bath, leading to a strong suppression of hyperfine coupling when the magnetic field is oriented in the plane of the qubit *g*-tensor. This understanding enables us to find an optimal operation regime that yields an improvement in spin coherence times of more than an order of magnitude compared with state of the art.

## Germanium two-qubit device

**Fig. 1:**
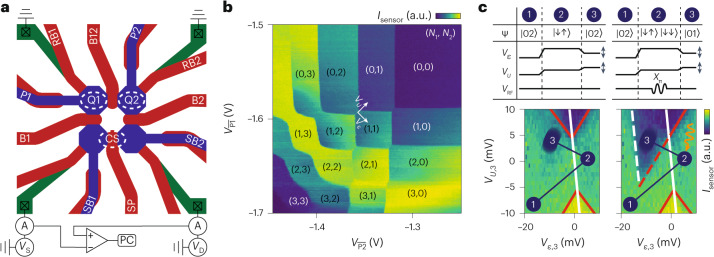
A germanium hole two-qubit system. **a**, Schematic drawing of the three-quantum-dot device. We define qubits Q1 and Q2 underneath plunger gates P1 and P2, respectively, that can be read out using the nearby charge sensor (CS) defined by gates SP, SB1 and SB2. The coupling between the qubits is controlled by B12, whereas the coupling of Q1 (Q2) to its respective reservoir is controlled by RB1 (RB2). We record the response of the charge sensor on the computer (PC) by measuring the differential current between the source (S) and drain (D) contacts, measured using transimpedance amplifiers A. *V*_S_, source bias voltage; *V*_D_, drain bias voltage. **b**, Two-quantum-dot charge stability diagram as a function of two virtualized plunger gate voltages ${V}_{\overline{{{{{\rm{P}}}}}{1}}}$ and ${V}_{\overline{{{{{\rm{P}}}}}{2}}}$, with the colour corresponding to the charge sensor current *I*_sensor_. The different charge configurations are indicated by the numbers in parentheses (*N*_1_, *N*_2_). The direction of the virtual detuning *ϵ* and on-site energy *U* axes are indicated. **c**, Spin-to-charge conversion is performed via latched Pauli spin blockade readout. The pulses applied to the *ϵ* and *U* axes, as well as the qubit drive pulses *V*_RF_, are shown in the top panels. The spin state ❘ψ❭ is initialized in the $\left\vert \downarrow \uparrow \right\rangle$ state by adiabatically sweeping across the interdot transition (1 → 2). Next we apply either no pulse (left panel) or an *X*_π_ pulse (right panel) to Q2 (2) and sweep (2 → 3) to the readout point (*V*_*ϵ*,3_, *V*_*U*,3_), which is rasterized to compose the entire map. Red lines indicate (extended) lead transition lines, whereas the white lines correspond to the interdot transition lines of the quantum-dot ground (solid) and excited (dashed) states.

We define a two-qubit system based on confined hole spins in a strained Ge/SiGe heterostructure quantum well^26^. The spins are confined in gate-defined quantum dots, formed respectively underneath plunger gates P1 and P2, with an additional gate B12 controlling the interdot coupling (Fig. 1a). In addition, we form a large quantum dot underneath gate SP to act as a charge sensor. Using two virtual gates $\overline{\,{{\mbox{P1}}}\,}$ and $\overline{\,{{\mbox{P2}}}\,}$ (Methods), we measure the charge stability diagram as plotted in Fig. 1b. Well-defined charge-occupancy regions can be observed, with the region in the top-right corner corresponding to both dots being fully depleted. We operate the device in the (1,1) charge regime and perform latched Pauli spin blockade readout^8,27,28^, as shown in Fig. 1c, where a distinct difference in the differential charge sensor current can be observed for the preparation of a $\left\vert \downarrow \downarrow \right\rangle$ and $\left\vert \downarrow \uparrow \right\rangle$ state.

## Heavy-hole *g*-tensor

**Fig. 2:**
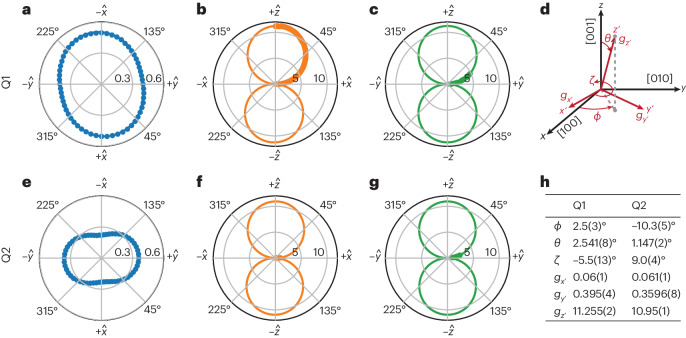
Measurement of the hole *g*-tensor. **a**–**c**,**e**–**g**, Cross-section of ${\overleftrightarrow{g}}$ of Q1 (**a**–**c**) and Q2 (**e**–**g**) in the *x*–*y* plane (**a**,**e**), *x*–*z* plane (**b**,**f**) and *y*–*z* plane (**c**,**g**) of the magnet frame. Dots indicate measurements of g* and the solid line corresponds to the fit of ${\overleftrightarrow{g}}$. Exemplary resonance spectra used to extract ${\overleftrightarrow{g}}$ are plotted in Supplementary Fig. 1. **d**, Diagram indicating the *zyz* Euler rotation angles *ϕ*, *θ* and *ζ* of the principle *g*-tensor axes **g**_*x*′_, **g**_*y*′_ and **g**_*z*′_. The approximate crystal directions are indicated in square brackets. **h**, Overview of the three *zyz* Euler angles *ϕ*, *θ* and *ζ* for the rotation of a *g*-tensor with principle components *g*_*x*′_, *g*_*y*′_ and *g*_*z*′_ for Q1 and Q2.

The confinement of holes in a two-dimensional strained germanium quantum well splits the heavy-hole (HH) and light-hole (LH) bands^11^. As the electrical confinement in the plane of the quantum well is notably weaker than the confinement in the growth direction, the hole wavefunction is expected to contain mostly heavy-hole components^11,22^. The degree of HH–LH mixing will affect the hole *g*-tensor, which is expected to be highly anisotropic for the heavy-hole states and much more isotropic for the light-hole states^29^. The general symmetric *g*-tensor can be described as a rotated diagonal 3 × 3 matrix ${\overleftrightarrow{g}}=R(\phi ,\theta ,\zeta )\,{{{\rm{diag}}}}\,({g}_{{x}^{{\prime} }},{g}_{{y}^{{\prime} }},{g}_{{z}^{{\prime} }}){R}^{-1}(\phi ,\theta ,\zeta\,)$, where *ϕ*, *θ* and *ζ* are Euler angles corresponding to successive intrinsic rotations around axes *zyz*, and *g*_*x*′_, *g*_*y*′_ and *g*_*z*′_ define the effective *g* factors along the *g*-tensor principle axes *x*′, *y*′ and *z*′, respectively (Fig. 2d). We reconstruct ${\overleftrightarrow{g}}$ for both Q1 and Q2 by measuring the effective *g* factor, g* = *hf*_Q_/(*μ*_B_*B*), where *h* is the Planck constant, *f*_Q_ = |**f**_Q_| is the qubit Larmor frequency and *μ*_B_ is the Bohr magneton, for different magnetic field orientations ${{{\bf{B}}}}=B\hat{{{{\bf{b}}}}}$. The measured data and fit of ${\overleftrightarrow{g}}$ are plotted in Fig. 2a–c,e–g for cuts through the *x*–*y*, *x*–*z* and *y*–*z* planes, respectively. The observed *g*-tensor is extremely anisotropic for both qubits, with *g*_*z*′_ ≈ 30*g*_*y*′_ ≈ 180*g*_*x*′_, and *g*_*z*′_ almost aligned to the sample growth direction *z*. The *g*-tensors of the two qubits are remarkably similar, with their principle axes lengths differing by less than 10%, the azimuth rotations *ϕ* and *ζ* by less than 15° and the elevation *θ* by less than 2° (Fig. 2h).

**Extended Data Fig. 1:**
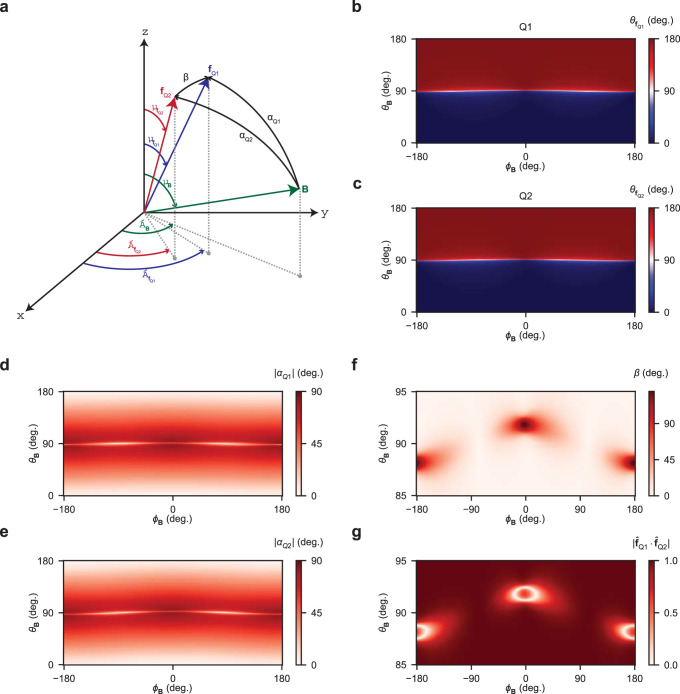
Projections of the fitted *g*-tensors. From the experimentally extracted the qubit *g*-tensors, we calculate the qubit quantisation axis $h{{{\bf{{f}}}_{{{{\rm{Q}}}}}}}={\mu }_{{{{\rm{B}}}}}{\overleftrightarrow{g}}\;{{{\bf{B}}}}$ as a function of **B**. **a**, Diagram illustrating the relevant angles. *θ*_**B**_, and *ϕ*_**B**_ are the elevation and azimuth angle of the applied magnetic field respectively. ${\theta }_{{{{\bf{{f}}}_{{{{\rm{Q}}}}i}}}}$, and ${\phi }_{{{{\bf{{f}}}_{{{{\rm{Q}}}}i}}}}$ are the elevation and azimuth angle of the resulting Larmor vector of Q*i*. *α*_*Qi*_ is the angle between the applied magnetic field and the resulting Larmor vector of Q*i* and *β* is the angle between the two quantisation axes of the two qubits. **b**,**c**, Elevation angle of the Larmor vector of Q1 (**b**) and Q2 (**c**), as a function of the orientation of the magnetic field. As a result of the large anisotropy of ${\overleftrightarrow{g}}$, the quantisation axis of the qubit rapidly flips from **f**_Q_∥*z* to **f**_Q_∥ −*z* as the magnetic field crosses the equator of the *g*-tensor. **d**,**e**, The absolute angle between the qubit quantisation axis and applied magnetic field direction $\left\vert {\alpha }_{Qi}\right\vert$ for Q1 (**d**) and Q2 (**e**). **f**, Angle between the qubit quantisation axes *β* as a function of the magnetic field orientation. Near the in-plane principle axis directions of the qubit *g*-tensors, a large misalignment between the two quantisation axes can be observed. **g**, Colour plot of $\left\vert {\hat{{{{\bf{f}}}}}}_{{{{\bf{Q1}}}}}\cdot {\hat{{{{\bf{f}}}}}}_{{{{\bf{Q2}}}}}\right\vert$, illustrating the orthogonality of the two qubit quantisation axes. For the ring-shaped regions where this quantity equals 0, around the *x* principle axis of the *g*-tensors, the qubit quantisation axes of Q1 and Q2 are perpendicular to each other.

Owing to the strong anisotropy in ${\overleftrightarrow{g}}$, the qubit quantization axis $h{{{{\bf{f}}}}}_{{{{\rm{Q}}}}}={\mu }_{{{{\rm{B}}}}}{\overleftrightarrow{g}}\ {{{\bf{B}}}}$ is not necessarily aligned with the applied magnetic field **B** as is the case in isotropic systems. In particular, any small deviation of **B** from the *x*′–*y*′ plane spanned by the two minor principal axes of ${\overleftrightarrow{g}}$ will result in a strong rotation of the qubit quantization axis towards $\pm \hat{z}$ (Extended Data Fig. 1b,c). Therefore, small variations between qubit *g*-tensors can lead to a sizeable difference in their quantization axes. Whereas the anisotropy between *g*_*z*′_ and *g*_*x*′,*y*′_ is expected from the quantum-well confinement, the additional in-plane anisotropy points to a non-circular confinement of the quantum dot, potentially caused by the interdot barrier breaking the individual quantum-dot symmetry. We suspect that the small but locally varying tilt of ${\overleftrightarrow{g}}$ with respect to the sample axes is caused by localized strain gradients as imposed by the nanostructured gate electrodes^24,30^.

## Charge noise

The connection between the confinement potential of the hole and LH–HH mixing gives rise to a sensitivity of ${\overleftrightarrow{g}}$ to local electric fields^23,29^. An electric field modulation will thus induce a variation $\delta\;{\overleftrightarrow{g}}$, leading to a modulation of the Larmor vector $h\delta {{{{\bf{f}}}}}_{{{{\rm{Q}}}}}={\mu }_{{{{\rm{B}}}}}\delta\;{\overleftrightarrow{g}}\;{{{\bf{B}}}}$. These modulations can be separated into changes parallel (longitudinal) or perpendicular (transverse) to the qubit quantization axis. The former will change the qubit energy splitting and provide a channel for dephasing due to, for example, charge noise^15^, whereas the latter enables driving the qubit through *g*-tensor magnetic resonance (*g*-TMR)^15,31,32^.

**Fig. 3:**
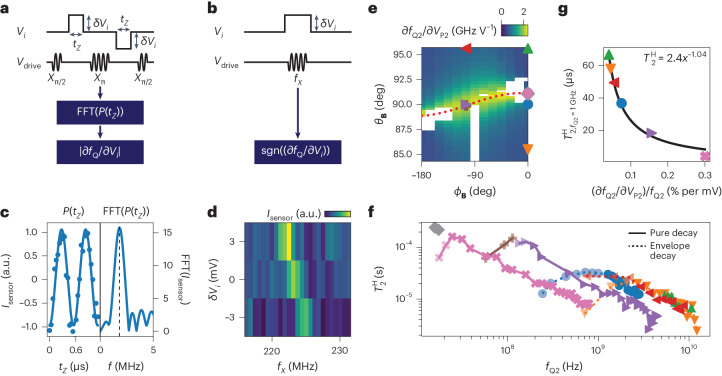
Electric field sensitivity and coherence dependence on magnetic field orientation. **a**, Pulse sequences used to measure the voltage sensitivity of the energy splitting ∂*f*_Q_/∂*V*_*i*_. A positive (negative) voltage pulse *δV*_*i*_ of varying length *t*_*Z*_ is applied to the test gate electrode *i* in the first (second) free evolution of a Hahn echo to extract ∣∂*f*_Q_/∂*V*_*i*_∣. **b**, Pulse sequences used to infer the sign of ∂*f*_Q_/∂*V*_*i*_ by assessing the shift in the qubit resonance frequency as a result of a voltage pulse *δV*_*i*_. **c**, Left: spin-up probability *P*(*t*_*Z*_) as reflected by the charge sensor current *I*_sensor_ as a function of the pulse length *t*_*Z*_, where the solid line is a fit to the data points. Right: fast Fourier transform (FFT) of *I*_sensor_, enabling the extraction of ∣∂*f*_Q_/∂*V*_*i*_∣. **d**, *I*_sensor_ as a function of the drive frequency *f*_*X*_ and *δV*_*i*_. The shift in the resonance frequency enables the sign of ∂*f*_Q_/∂*V*_*i*_ to be extracted. **e**, The qubit energy splitting sensitivity to a voltage change on the plunger gate ∂*f*_Q2_/∂*V*_P2_, as a function of different magnetic field orientations *ϕ*_**B**_ and *θ*_**B**_. *B* is adapted to keep *f*_Q2_ constant at *f*_Q2_ = 1.36(7) GHz. Data acquisition is hindered for the white areas as a result of limited qubit readout or addressability for these magnetic field orientations (the exact filed orientations of which are given in Supplementary Table 1). **f**, Hahn coherence time ${T}_{2}^{\,{\rm{H}}}$ as a function of the qubit frequency *f*_Q2_, for different magnetic field orientations indicated by the coloured markers in **e**. Solid lines correspond to ${T}_{2}^{\,{\rm{H}}}$ as extracted from a pure decay, whereas dotted lines correspond to ${T}_{2}^{\,{\rm{H}}}$ as extracted from the envelope of the nuclear spin-induced collapse and revival. Data indicated by opaque markers are used to fit the power law dependence of ${T}_{2}^{\,{\rm{H}}}$. **g**, Expected ${T}_{2,{\,{{f}}}_{{{{\rm{Q}}}}2}\, =\, 1\,{{{\rm{GHz}}}}}^{\,{\mathrm{H}}}$ as extracted from a power law fit to the opaque data markers in **f** as a function of the gate voltage sensitivity (∂*f*_Q2_/∂*V*_P2_)/*f*_Q2_ from **e**. The coloured markers correspond to the different magnetic field orientations as indicated in **e**. The solid black line is a fit of ${T}_{2}^{{\mathrm{H}}}=a{x}^{\beta }$ to the data, yielding a scaling factor of *a* = 2.4 and an exponent of *β* = −1.04(8).

First, we focus on the longitudinal electric field sensitivity and measure ∂*f*_Q2_/∂*V*_*i*_ of Q2 for a potential applied to gate electrode *i*. We determine the change in qubit frequency *f*_Q_ from the acquired phase in a Hahn echo experiment, when applying a small voltage pulse *δV*_*i*_ during the evolution time^15,32^ (Fig. 3a–d and Methods). Figure 3e shows ∂*f*_Q2_/∂*V*_P2_ for different magnetic field orientations, for a fixed *f*_Q2_ = 1.36(7) GHz. We observe the qubit energy splitting to be most sensitive to electric field fluctuations when **B** aligns to the *x*′–*y*′ plane (indicated by the red dotted line), with ∂*f*_Q2_/∂*V*_P2_ > 2 GHz V^−1^.

**Extended Data Fig. 2:**
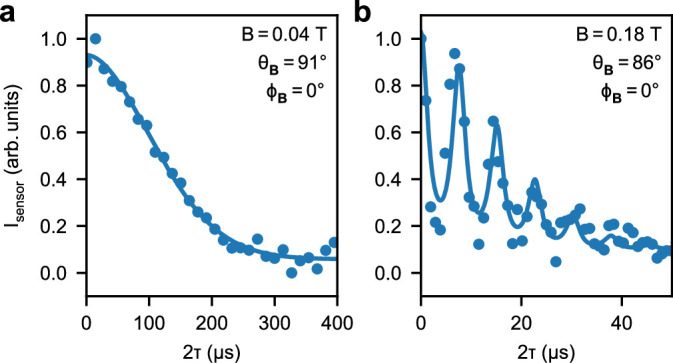
Hahn echo decay of Q2. **a**, Hahn echo decay of Q2, for the magnetic field aligned with the hyperfine sweet plane. Solid line is a fit to the data used to extract ${T}_{2}^{\,{\rm{H}}}$. **b**, Hahn echo decay of Q2, for the magnetic field aligned away from the hyperfine sweet plane. A collapse-and-revival structure can be observed due to the interaction of the qubit with the precession of the ^73^Ge nuclear spins. Solid line is a fit to the data used to extract ${T}_{2}^{\,{\rm{H}}}$.

**Extended Data Fig. 3:**
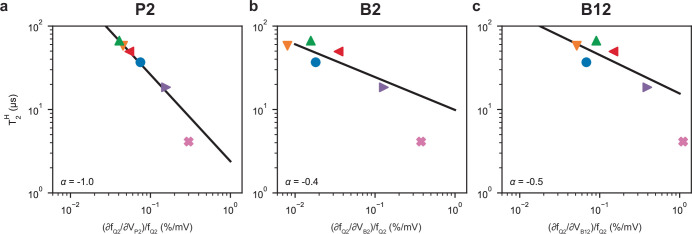
Q2 coherence as a function of the different gate voltage sensitivities. **a**–**c**, Observed Hahn echo coherence time (data from Fig. 3g in the main text), as a function of the relative qubit frequency sensitivity (∂*f*_Q2_/∂*V*_*i*_)/*f*_Q2_ to a voltage fluctuation on gate P2 (**a**), B2 (**b**), or B12 (**c**). Voltage sensitivities are obtained from the data for gate P2 (Fig. 3e) from the fits of $\delta\;{\overleftrightarrow{g}}$ presented in Fig. 4 of the main text, as well as Extended Data Fig. 4 for gates B2 and B12. Black lines are fits to a power law ${T}_{2}^{\,{H}}=a\exp ({((\partial {f}_{{{{\rm{Q}}}}2}/\partial {V}_{i})/{f}_{{{{\rm{Q}}}}2})}^{\alpha })$, with *a* a scaling factor and *α* the exponent. We find the correct trend with *α* = − 1 (see Methods) for a voltage noise effectively coming from the plunger gate of the qubit, indicating the dominant charge traps are located directly above our qubit for most magnetic field orientations. For very specific field orientations where the qubit sensitivity to noise from other directions is particularly strong, such as for the pink data point, differently located charge traps could limit ${T}_{2}^{\,{H}}$.

If qubit decoherence is limited by fluctuations in ${\overleftrightarrow{g}}$ induced by charge noise, we expect the size of the qubit frequency fluctuations *δf*_Q_ to increase linearly with *B* and to depend strongly on the orientation of **B** as governed by the corresponding longitudinal electric field sensitivity. To verify this, we perform a Hahn echo experiment and extract the echo coherence times ${T}_{2}^{\,{\rm{H}}}$ by fitting the data to an envelope exponential decay, excluding nuclear spin effects (Extended Data Fig. 2 and Methods). In Fig. 3f we plot ${T}_{2}^{\,{\rm{H}}}$ as a function of the qubit frequency (obtained by varying *B*) for different orientations of **B** as indicated by the coloured markers in Fig. 3e. For a large enough *B*, we observe a power law dependence of ${T}_{2}^{\,{\rm{H}}}\propto {f_{{{\rm{Q}}}}}^{{-1}}$, consistent with a 1/*f* charge noise spectrum acting on the qubit^15,33^ (see Methods). We note that for small *B*, the finite spread of the precession frequencies of the nuclear spin ensemble limits qubit coherence, resulting in a sharp decrease^34^ in the extracted ${T}_{2}^{\,{\rm{H}}}$. Next, we correlate the charge-noise-limited ${T}_{2}^{\,{\rm{H}}}$ at *f*_Q2_ = 1 GHz to the longitudinal electric field sensitivity on gate P2 for different orientations of **B** (Fig. 3g). The good fit to a power law with an exponent of −1 confirms that the main sources of charge noise are located directly above the qubit near gate P2 (the field sensitivity correlation for other gates is shown in Extended Data Fig. 3).

**Fig. 4:**
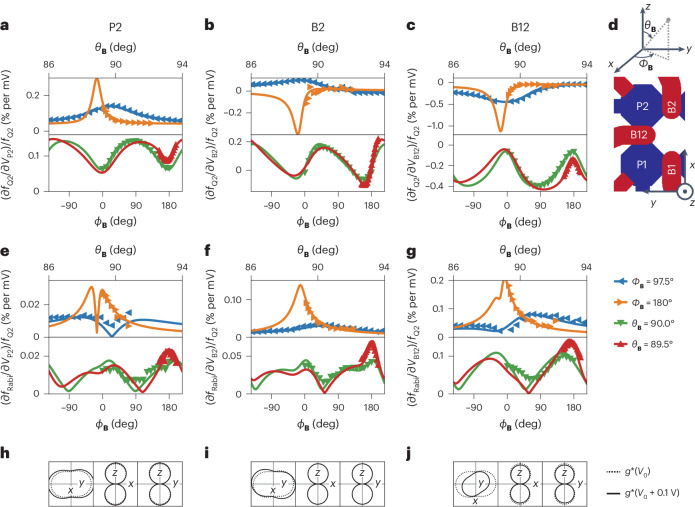
Reconstruction of $\partial\;{\overleftrightarrow{g}}/\partial {V}_{i}$ for differently oriented electrostatic gates. **a**–**c**, Relative voltage sensitivity of the energy splitting (∂*f*_Q2_/∂*V*_*i*_)/*f*_Q2_ of Q2 for a voltage excitation applied to gates P2 (**a**), B2 (**b**) and B12 (**c**). Top panels correspond to sweeps of the magnetic field elevation *θ*_**B**_, whereas bottom panels correspond to sweeps of the in-plane angle *ϕ*_**B**_. The solid lines correspond to projections of the $\partial\;{\overleftrightarrow{g}}/\partial {V}_{i}$ fitted to the data. **d**, Schematic illustration of the qubit layout indicating the different electrostatic gates. **e**–**g**, Relative Rabi frequency of (∂*f*_Rabi_/∂*V*_*i*_)/*f*_Q2_ of Q2 for a drive voltage excitation *V*_*i*_ applied to gates P2 (**e**), B2 (**f**) and B12 (**g**). Solid lines correspond to the projection of the $\partial\;{\overleftrightarrow{g}}/\partial {V}_{i}$ as fitted to the data in panels **a**–**c**. **h**–**j**, Cross-section of the change in ${\overleftrightarrow{g}}$ in the *x–y*, *x*–*z* and *y*–*z* planes of the magnet frame when applying a voltage pulse of 0.1 V on gates P2 (**h**), B2 (**i**) and B12 (**j**), with respect to the normal operation voltage *V*_0_. Dotted lines correspond to the cross-sections of ${\overleftrightarrow{g}}$, whereas solid lines represent $$\begin{array}{l}{\overleftrightarrow{g}}+\delta\;\,{\overleftrightarrow{g}}_{{i}}\,\left(0.1\,{\mathrm{V}}\right)\end{array}$$.

To gain a complete understanding of the mechanism underlying the electric modulation of ${\overleftrightarrow{g}}$, we reconstruct $\partial\;{\overleftrightarrow{g}}/\partial {V}_{i}$ for Q2 and for *i* = P2, B2 and B12 (see Fig. 4d for the relative orientation of the three gates). We measure (∂*f*_Q2_/∂*V*_*i*_)/*f*_Q2_ for different magnetic field orientations, enabling $\partial\;{\overleftrightarrow{g}}/\partial {V}_{i}$ to be extracted (Methods). All measurements are performed at constant *f*_Q2_ = 225 MHz and we show the relative electric potential sensitivity and corresponding fits in Fig. 4a–c. The extracted parameters that describe the *g*-tensor modulation are detailed in Extended Data Table 1. To illustrate what happens to ${\overleftrightarrow{g}}$ as the gates are pulsed, we sketch cross-sections of ${\overleftrightarrow{g}}$ and $$\begin{array}{l}{\overleftrightarrow{g}}+\delta\;{\overleftrightarrow{g}}_{{{i}}}\;\left(100\,{\mathrm{mV}}\right)\end{array}$$ in Fig. 4h–j. The plunger gate directly above the qubit mostly scales the *g*-tensor principle axes (‘breathing’), while the neighbouring barrier gates also induce a rotation of ${\overleftrightarrow{g}}$.

**Extended Data Fig. 4:**
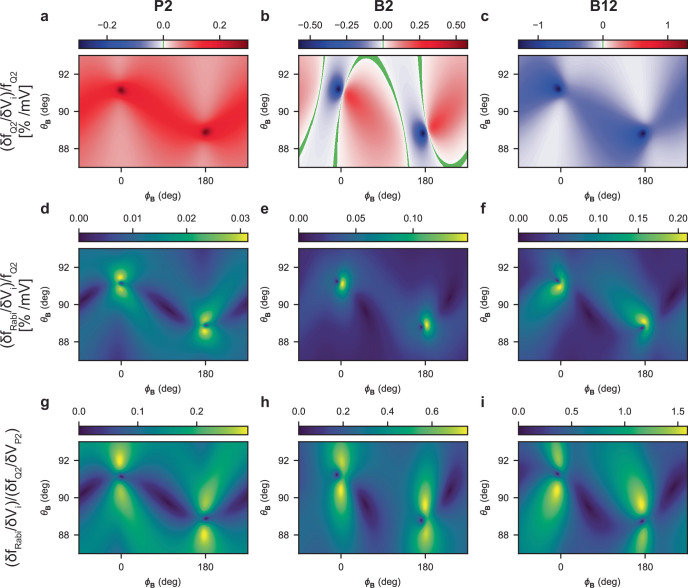
Longitudinal and transverse components of the fitted $\partial\;{\overleftrightarrow{g}}/\partial {V}_{i}$. **a**–**c**. Using the fitted $\partial\;{\overleftrightarrow{g}}/\partial {V}_{i}$, as detailed in Fig. 4 of the main text and Extended Data Table 1, we plot the expected normalized resonance frequency fluctuation of Q2 as a result of a voltage fluctuation on gate P2 (**a**), B2 (**b**), and B12 (**c**) for different magnetic field orientations. Zero crossings are marked in green, to indicate the presence of a true sweet spot. **d**–**f**. Expected normalized Rabi frequency fluctuation of Q2 as a result of a drive excitation with amplitude *V*_*i*_ on gate P2 (**d**), B2 (**e**), and B12 (**f**) for different magnetic field orientations. **g**–**i**. Expected ratio of the transverse and longitudinal components (∂*f*_Rabi_/∂*V*_*i*_)/(∂*f*_Q2_/∂*V*_P2_) as a result of a drive amplitude on gate P2 (**g**), B2 (**h**), and B12 (**i**) for different magnetic field orientations. We assume the noise to couple in predominantly as if it is applied to the plunger gate P2.

A true sweet spot to noise originating near gate *i* exists when ∂*f*_Q_/∂*V*_*i*_ = 0. We only find such zero crossings for potentials applied to side gate B2, as visible in Fig. 4b (full *θ*_**B**_–*ϕ*_**B**_ projections are shown in Extended Data Fig. 4). For voltage fluctuations applied to gates P2 and B12, we find that a reduction of the electric field sensitivity is possible, but no true sweet spot exists for any (*θ*_**B**_, *ϕ*_**B**_). These effects are dominated by the dynamic tilting of ${\overleftrightarrow{g}}$, which we believe to be caused by the hole wavefunction moving in a local strain gradient^24,30,35^, not taken into account by previous models^22^.

Whereas the longitudinal component of the *g*-tensor modulation leads to decoherence, the transverse part enables an electric drive of the qubit through *g*-TMR. Therefore, our reconstruction of $\partial\;{\overleftrightarrow{g}}/\partial {V}_{i}$ enables us to compare the expected Rabi frequency from *g*-TMR with the observed Rabi frequency. We measure the angular dependence of the Rabi frequency *f*_Rabi_ of the qubit, for a resonant electric drive with amplitude *V*_*i*_ applied to gate P2, B2 or B12 and extract (∂*f*_Rabi_/∂*V*_*i*_)/*f*_Q2_. The results, shown in Fig. 4e–g, reveal a striking agreement between the measured and expected Rabi frequency due to the *g*-TMR (Methods). The agreement between the data and the projection of $\partial\;{\overleftrightarrow{g}}/\partial {V}_{i}$, both in absolute size and magnetic field dependence, confirms that the main driving mechanism of planar germanium hole qubits is *g*-TMR.

## Hyperfine interaction

Our qubits are defined in a natural germanium quantum well, where ^73^Ge is the only isotope with non-zero nuclear spin. As a result, the hole wavefunction overlaps with ~10^6^ nuclear spins (Methods), leading to a fluctuating Overhauser field acting on the hole spin. The contributions of the Overhauser field can be separated into longitudinal and transverse components with respect to the quantization axis of the nuclear spins^34^. Whereas temporal fluctuations of both components can lead to qubit dephasing, longitudinal field fluctuations are mainly caused by the quasi-static dipole–dipole interaction between nuclear spins^36,37^ and can easily be echoed out. However, the transverse part contains a spectral component at the Larmor frequency of the nuclear spins, which leads to a collapse and revival of coherence when performing spin-echo experiments, as predicted previously^36,38^ and observed in gallium arsenide^34^ and germanium^39^.

**Fig. 5:**
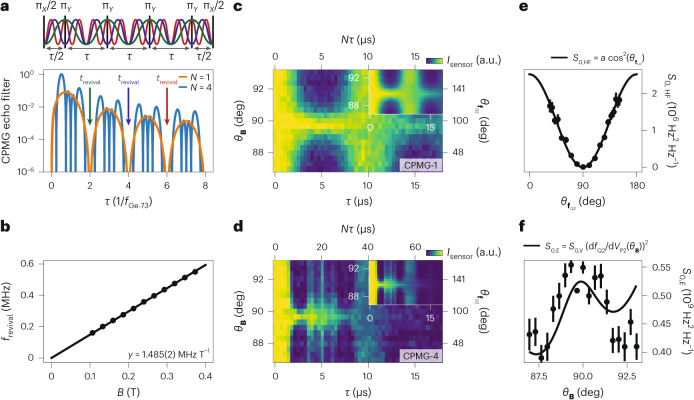
Collapse and revival of qubit coherence due to hyperfine interaction. **a**, Filter function of the CPMG pulse sequence, for *N* = 1 and 4 decoupling pulses, illustrating full suppression of noise with a characteristic frequency *f* = *n*/(2*τ*), where *n* is any integer. **b**, Extracted revival frequency *f*_revival_ as a function of the magnetic field strength *B* (full data shown in Extended Data Fig. 6). We extract a gyromagnetic ratio of the ^73^Ge nuclear spin of *γ* = 1.485(2) MHz T^−1^. **c**,**d**, Normalized charge sensor signal *I*_sensor_ for a CPMG sequence with 1 (**c**) and 4 (**d**) decoupling pulses as a function of the spacing between two subsequent decoupling pulses *τ* and *θ*_**B**_. *Nτ* is the total evolution time. *ϕ*_**B**_ = 97.5° and *B* = 133 mT. The inset for each displays the fit to the data from which we extract *S*_0,HF_(*θ*_**B**_) and *S*_0,E_(*θ*_**B**_) (Methods). **e**, The extracted strength of the hyperfine interaction *S*_0,HF_ as a function of ${\theta }_{{{{{\bf{f}}}}}_{{{{\rm{Q}}}}}}$. The black line is a fit of the data to $a\,{\cos }^{2}({\theta }_{{{{{\bf{f}}}}}_{{{{\rm{Q}}}}}})$, with *a* = 2.5 × 10^6^ Hz^2^ Hz^−1^. Data are presented as the fitted values of *S*_0,HF_(*θ*_**B**_), with error bars indicating the 1 s.d. uncertainty of the fit. **f**, The extracted strength of the 1/*f* electric field noise at 1 Hz *S*_0,E_. The black line is a fit of the data to ${S}_{0,{{{\rm{E}}}}}={S}_{0,{{{\rm{V}}}}} {(\partial {f}_{{{{\rm{Q}}}}2}/\partial {V}_{{{{\rm{P}}}}2}({\theta }_{{{{\bf{B}}}}}))}^{2}$, where ∂*f*_Q2_/∂*V*_P2_(*θ*_**B**_) is the electric field sensitivity of the qubit frequency to the top gate voltage as extracted from Fig. 4 and the effective voltage noise *S*_0,V_ = 6.1 × 10^−10^ V^2^ Hz^−1^ the only fit parameter. Data are presented as the fitted values of *S*_0,E_(*θ*_**B**_), with error bars indicating the 1 s.d. uncertainty of the fit.

**Extended Data Fig. 5:**
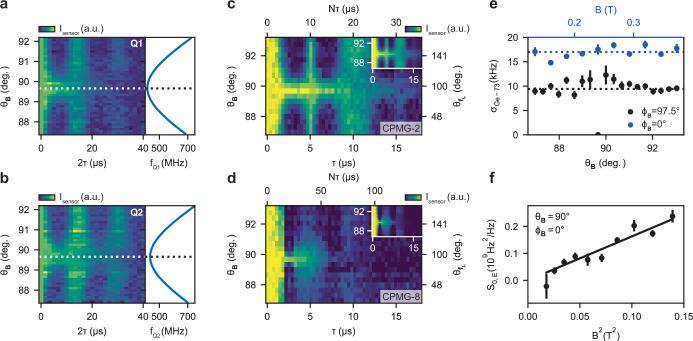
Hyperfine interaction between the qubits and the ^73^Ge nuclear spins. **a**,**b**, Normalized charge sensor signal for a Hahn echo experiment as a function of the total free evolution time 2*τ* (colour map in left subpanel) and the qubit frequency *f*_Q*i*_ (plot in right subpanel) as a function of the elevation angle of the magnetic field for Q1 (**a**) and Q2 (**b**). *B* = 89 mT is kept constant throughout the measurement. For any *θ*_**B**_ away from the ${x}^{{\prime} }{y}^{{\prime} }$ plane of ${\overleftrightarrow{g}}$, we observe the collapse-and-revival characteristic for a well-defined spectral component acting on the qubit. Due to the small tilt between the two qubit *g*-tensors, a common hyperfine sweet spot is defined by the intersection of two differently tiled ellipsoids in the lab frame, at *ϕ*_**B**_ = 97.5°, *θ*_**B**_ = 89.7°. Selecting this magnetic field orientation allows to operate both qubits in their respective hyperfine sweet planes simultaneously. Here, we find ${T}_{2,{{{\rm{Q}}}}1}^{\,{H}}=8.1(2)\,\mu {{{\rm{s}}}}$ and ${T}_{2,{{{\rm{Q}}}}2}^{\,{H}}=11.5(6)\,\mu {{{\rm{s}}}}$ for **B** as specified. **c**,**d**, Normalized charge sensor signal for a CPMG sequence with respectively 2 (**c**), and 8 (**d**) decoupling pulses, as a function of the spacing between two subsequent decoupling pulses *τ* and *θ*_**B**_. *Nτ* is the total evolution time. The magnetic field strength is *B* = 133 mT and the azimuth angle is *ϕ*_**B**_ = 97.5°. The inset displays the fit to the data. These data complement the dataset displayed in Fig. 5c,d of the main text. **e**, Width of the hyperfine line *σ*_Ge-73_ as extracted from the CPMG coherence as a function of *B* (Extended Data Fig. 6, *ϕ*_**B**_ = 0°, blue markers), and as extracted from the CPMG coherence as a function of *θ*_**B**_ (Fig. 5c,d of the main text, *ϕ*_**B**_ = 97.5°, black markers). Data are presented as the fitted values of *σ*_Ge-73_ with error bars indicating the 1*σ* uncertainty of the fit. We find *σ*_Ge-73_ to be independent of *θ*_**B**_ in the experimental range, but observe an offset for the two different azimuth directions. Dashed lines correspond to the average ${\overline{\sigma }}_{{{{\rm{Ge}}}}{{\mbox{-}}}73}=9.4$ kHz for *ϕ*_**B**_ = 97.5° and ${\overline{\sigma }}_{{{{\rm{Ge}}}}{{\mbox{-}}}73}=17$ kHz for *ϕ*_**B**_ = 0°. **f**, The intensity of the 1/*f* component of the spectrum at 1 Hz extracted from the data presented in Extended Data Fig. 6. Data are presented as the fitted values of *S*_0,E_ with error bars indicating the 1 s.d. uncertainty of the fit. We find ${S}_{0,{{{\rm{E}}}}}\propto {B}^{2}\propto {f}_{{{{\rm{Q}}}}2}^{\,{2}}$, as expected for charge noise with a 1/*f* spectrum.

**Extended Data Fig. 6:**
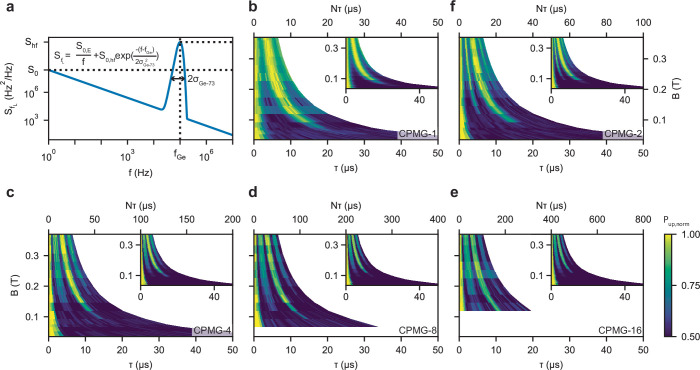
CPMG dynamical decoupling as a function of magnetic field strength. **a**, Schematic illustrating the power spectrum of noise assumed for the fitting. The spectrum consists of a 1/*f* charge noise component with a power *S*_0,E_ at 1 Hz, plus a Gaussian spectral line at *f*_Ge-73_ with width *σ*_Ge-73_ and power *S*_0,hf_. **b**–**f**, Normalized charge sensor signal *I*_sensor_ for a CPMG sequence with 1 (**b**), 2 (**c**), 4 (**d**), 8 (**e**), and 16 (**f**) decoupling pulses respectively, as a function of the spacing between two subsequent decoupling pulses *τ* and *B*. *Nτ* is the total evolution time. *ϕ*_**B**_ = 0° and *θ*_**B**_ = 90°. The inset displays the fit to the data from which we extract *γ*_Ge-73_ = 1.48 MHz/T and *σ*_Ge-73_ = 17 kHz. The extracted charge-induced noise spectrum *S*_0,E_(*B*) is plotted in Extended Data Fig. 5f.

The hyperfine interaction between heavy-hole states and nuclear spins is expected to be highly anisotropic^16^, unlike the isotropic contact hyperfine interaction observed for conduction-band electrons. In fact, for the ^73^Ge isotope, the Ising term (out of plane, ∝*s*_*z*_*I*_*z*_) is numerically estimated to be ~50 times larger than the in-plane (∝*s*_*x*_*I*_*x*_, *s*_*y*_*I*_*y*_) components^40^, with *s*_*i*_ and *I*_*i*_ the *i* component of the pseudospin-1/2 and nuclear spin operators, respectively. As a result, hyperfine interaction between the heavy hole and the surrounding nuclear spin bath is expected to be negligible for an in-plane magnetic field^16,40^. To study the hyperfine anisotropy for planar germanium qubits, we perform a Carr–Purcell–Meiboom–Gill (CPMG) experiment, which constitutes an effective bandpass filter for the noise acting on the qubit with a frequency *f* = 1/*τ* set by the free evolution time *τ* between the *Y*_π_-pulses (Fig. 5a). We apply CPMG sequences with *N* = 1, 2, 4 and 8 decoupling pulses to Q2 and measure the spin state as a function of *τ*, as shown in Fig. 5c,d for *N* = 1 and *N* = 4, respectively (data for *N* = 2 and *N* = 8 is shown in Extended Data Fig. 5c,d, respectively). We observe the expected collapse and revival of the coherence and find *f*_revival_ = *γ*∣**B**∣ with *γ* = 1.485(2) MHz T^−1^ (Fig. 5b and Extended Data Fig. 6), in good agreement with the gyromagnetic ratio of the ^73^Ge nuclear spin *γ*_Ge-73_ = 1.48 MHz T^−1^.

**Extended Data Fig. 7:**
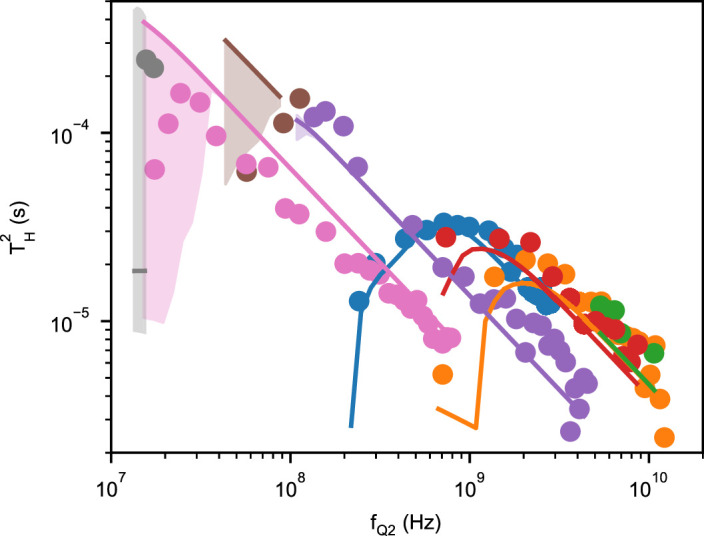
Expected qubit coherence from extracted noise parameters. We simulate the expected CPMG-1 decay using the filter formalism as detailed in the Methods section, using the noise power parameters as extracted from the CPMG experiment displayed in Fig. 5 of the main text: $\sqrt{{S}_{V}}=24.7\,\mu {\rm{V}}/\sqrt{\,{{\mbox{Hz}}}\,},{\gamma }_{{{{\rm{Ge}}}}{{\mbox{-}}}73}=1.48 \;{{\rm{MHz}}/{\mathrm{T}}}$, ${S}_{0,{{{\rm{hf}}}}}=2.5\times 1{0}^{12}{\cos }^{2}({\theta }_{{f}_{{{{\rm{Q}}}}2}}),{\sigma }_{{{{\rm{Ge}}}}{{\mbox{-}}}73}$ $=17 \;{\rm{kHz}}$ for *ϕ*_**B**_ = 0°, and *σ*_Ge-73_ = 9 kHz for *ϕ*_**B**_ = −105°. We then fit the simulated decay using the same procedure as used in Fig. 3f of the main text to extract the envelope ${T}_{2}^{\,{\rm{H}}}$. Markers indicate experimental data (reproduced from Fig. 3) and the solid lines correspond to the envelope decay time as predicted by the model. The excellent agreement between the simulation and data, without the need for any fitting parameters, confirms our understanding of the system. The 1/*f*_Q2_ decay of the envelope coherence can be explained by an effective voltage noise on plunger gate P2, while the low-*B* drop-off is caused by the finite spread of the ^73^Ge precession frequencies. The shaded area indicates the uncertainty in ${T}_{\,{\rm{H}}}^{2}$, given an uncertainty of ± 20 *μ*T in the *z*-component of the magnetic field. For very small *B*, this yields a significant uncertainty in *θ*_**B**_, thus complicating an accurate prediction of ${T}_{\,{\rm{H}}}^{2}$.

We fit the spectroscopy data (insets of Fig. 5c,d) using the filter formalism^33,41^ and assuming a noise spectrum ${S}_{{f}_{Q}}$ acting on the qubit that consists of a 1/*f* part caused by charge noise and a sharp spectral component at the precession frequency of the ^73^Ge nuclear spins (details in Methods). This enables us to extract the intensity of the nuclear noise *S*_0,HF_(**B**) as well as the charge noise *S*_0,E_(**B**) components. Figure 5e shows *S*_0,HF_ as a function of the elevation of the Larmor vector ${\theta }_{{{{{\bf{f}}}}}_{{{{\rm{Q}}}}2}}=\arccos \left(\frac{{{{\bf{{f}}}_{{{{\rm{Q}}}}2}}}\cdot \hat{z}}{{f}_{{{{\rm{Q}}}}2}}\right)$. We find that the data closely follows a relation ${S}_{0,{{{\rm{HF}}}}}\propto {\cos }^{2}\left({\theta }_{{{{{\bf{f}}}}}_{{{{\rm{Q}}}}2}}\right)$, providing strong experimental evidence of the predicted Ising coupling^16,40,42^. As a result, there exists a sweet plane approximately spanned by the ${x}^{{\prime} }{y}^{{\prime} }$ axes of ${\overleftrightarrow{g}}$, where the qubit is mostly insensitive to nuclear spin noise. The finite width of the hyperfine distribution of *σ*_Ge-73_ = 9–16 kHz leads to an unrecoverable loss of qubit coherence for small *B*, as seen in Fig. 3f and Extended Data Fig. 6. Next, using the extracted noise spectrum parameters, we model the Hahn coherence time as a function of **B** and find a good agreement with the data (Extended Data Fig. 7).

**Extended Data Fig. 8:**
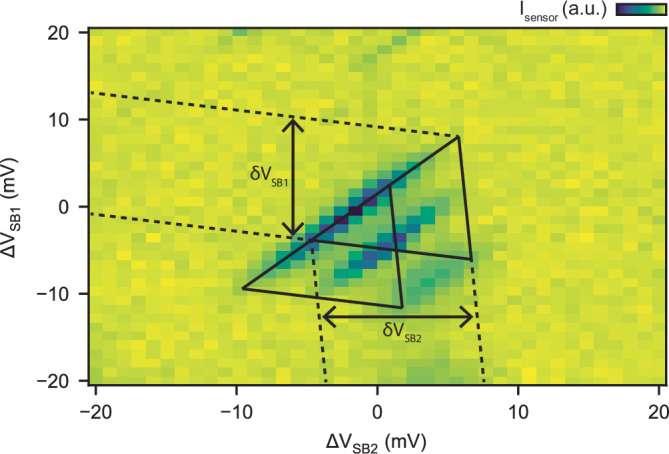
Lever arm extraction of the plunger gates. We extract the lever arm of the plunger gates by tuning the charge sensor into a transport double quantum dot underneath gates SB1 and SB2, which are lithographically identical to qubit gates P1 and P2. We measure the bias triangles at *V*_SD_ = 1 mV and find an average plunger lever arm of *α*_P_ = 7.4(8)%, with the uncertainty as obtained from the scatter of *α* for gates SB1 and SB2.

Finally, assuming all charge noise to originate near P2, we convert *S*_0,E_(**B**) to an effective electrical noise power (Fig. 5f) and find a value for the effective voltage noise of *S*_V_ = 610 μV^2^ Hz^−1^ at 1 Hz, corresponding to an effective voltage noise of 25 μV Hz^−1/2^ on P2. Using the estimated plunger gate lever arm *α*_P_ = 7.4% (Extended Data Fig. 8), we extract a charge noise of 1.9 μeV Hz^−1/2^, in good agreement with charge noise measurements on similar devices^12^.

## Sweet-spot operation

The detailed understanding of the hole qubit coherence for different magnetic field orientations enables an optimal operation regime to be selected. For any magnetic field orientation away from the hyperfine sweet plane, nuclear spin noise limits qubit coherence in natural germanium samples. However, the slight but notable tilt between the two qubit *g*-tensors limits this further to a single spot where the two circles intersect: *ϕ*_**B**_ = 97.5° and *θ*_**B**_ = 89.7° for this device (Extended Data Fig. 5a,b). The existence of such common hyperfine sweet spots is not guaranteed for larger qubit systems when the individual qubit *g*-tensors differ slightly. Furthermore, we observe that this hyperfine sweet plane coincides with the hotspots for charge-induced decoherence (Extended Data Fig. 4), preventing full use of charge noise sweet spots. In fact, we estimate charge-noise-limited coherence times and quality factors to be improved by about an order of magnitude for optimal magnetic field orientations. This underlines the need for isotopically purified materials, despite the Ising-type hyperfine interaction of the heavy hole.

**Fig. 6:**
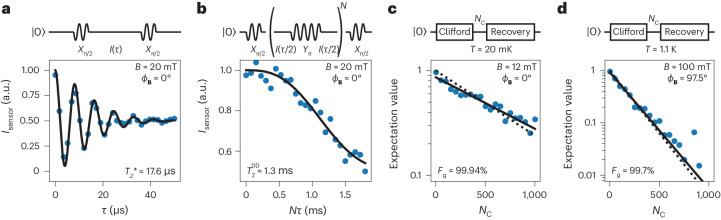
Coherence figures at low magnetic field in the hyperfine sweet spot. **a**, Free induction decay coherence as measured through a Ramsey experiment. The data constitute of an average of ten traces, for a total integration time of approximately 5 min (12 h dataset, Extended Data Fig. 9), and we find a coherence time of ${T}_{2}^{* }$ = 17.6 μs. **b**, CPMG dynamical decoupling coherence, as measured for a sequence with 250 refocusing pulses. We find a coherence time of ${T}_{2}^{{{{\rm{DD}}}}}$ = 1.3 ms. **c**, Randomized benchmarking of the performance of Q2, at base temperature *T* = 20 mK. The solid line is a fit of the data to *P* = *a* exp(−(1 − 2*F*_C_)*N*_C_), with *a* the visibility, *F*_C_ the Clifford gate fidelity, and *N*_C_ the number of Clifford gates, from which we extract a single-qubit gate fidelity of *F*_g_ = 99.94%. The reduced visibility for larger *N*_C_ is caused by the readout being affected by the large number of pulses applied to the gate, but does not affect the extracted fidelity (Methods), as indicated by the dotted line where we fix *a* = 1. **d**, Randomized benchmarking at a fridge temperature of *T* = 1.1 K for Q2. We now operate in the joint Q1–Q2 hyperfine sweet spot at *ϕ*_**B**_ = 97.5°. We extract a single-qubit gate fidelity of *F*_g_ = 99.7%.

**Extended Data Fig. 9:**
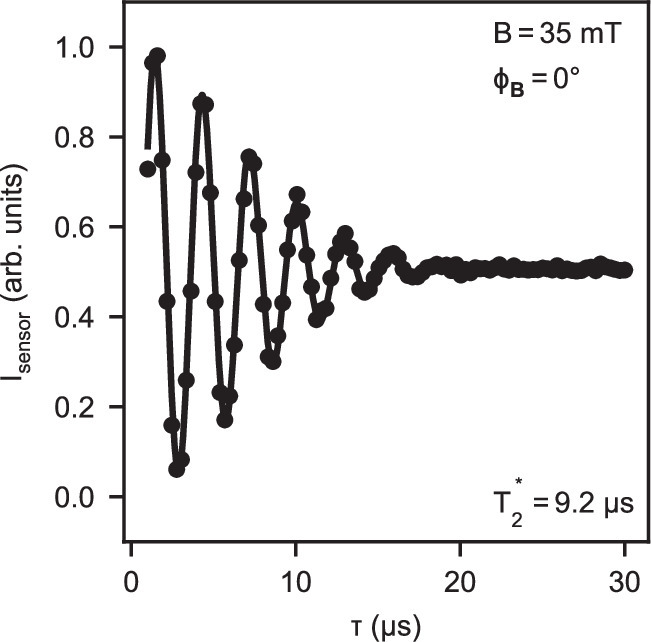
Extended Ramsey measurement at *B* = 35 mT. Normalized charge sensor signal as a function of the waiting time *τ* for a Ramsey experiment. The data constitute of an average of 750 traces, for a total integration time of over 12 hours and we find a coherence time of ${T}_{2}^{\,{*}}=9.2\,\mu {{{\rm{s}}}}$.

For our device, we aim to optimize the coherence of Q2 by lowering the magnetic field strength and operate along the hyperfine sweet plane of Q2, with *ϕ*_**B**_ = 0° to strike a balance between low charge noise sensitivity and high operation speed. We first assess the free induction decay coherence time by performing a Ramsey experiment (Fig. 6a and Extended Data Fig. 9). We set *B* = 20 mT, such that *f*_Q2_ ≈ 21 MHz and *f*_Rabi_ = 1 MHz and find ${T}_{2}^{\;* }=17.6\,\upmu{\mathrm{s}}$, which is about an order of magnitude larger than shown previously for germanium hole qubits^39^. We can further extend the coherence using dynamical decoupling and find coherence times (${T}_{2}^{\,{\mathrm{DD}}}$) beyond 1 ms (Fig. 6b). Operation at low magnetic field also has implications for the speed of single-qubit operations, as these are expected and observed to scale with *B*. The single-qubit gate performance is ultimately governed by the ratio of the operation and coherence time and should thus be preserved at low magnetic fields. We confirm this by performing randomized benchmarking, using a Clifford group based on *X*_π_ and *X*_π/2_ pulses and virtual *Z* updates (Supplementary Table 2). We find an optimal average single-qubit gate fidelity *F*_g_ (with 0.875 physical gates per Clifford) of 99.94% at *B* = 12 mT (Fig. 6c). Furthermore, we find that the fidelity remains well above 99% when operating our qubits at an elevated temperature of *T* = 1.1 K, where more cooling power is available (Fig. 6d). Lowering the qubit frequency thus provides the opportunity to increase qubit coherence while maintaining a high single-qubit gate performance. This provides an avenue to improve two-qubit gate performance, which has typically been limited by the comparatively short coherence time of the germanium hole qubit^8,9^.

## Conclusions

In summary, we report a fully electrically controlled two-qubit system defined by single hole spins in a strained germanium quantum well. The hole *g*-tensor of both qubits is characterized, revealing a strong anisotropy with respect to the heterostructure growth direction. The two qubit *g*-tensors are remarkably similar and vary by less than 10%, indicative of a high degree of uniformity of the electrostatic confinement. However, the small tilt (*δθ* ≈ 1°) combined with the large anisotropy of ${\overleftrightarrow{g}}$ leads to measurable effects, in particular for magnetic field orientations in proximity to the *g*-tensor minor principle axes. The slight tilt of ${\overleftrightarrow{g}}$ is probably the result of local strain gradients and could thus be controlled through material and gate stack optimization or by modifying the LH–HH mixing, defined by material stoichiometry^43^ and quantum-dot confinement^21^.

The *g*-tensor anisotropy is also reflected in the qubit sensitivity to electric field fluctuations. We find that ${\overleftrightarrow{g}}$ breathes and tilts under electric field fluctuations, leading to charge-noise-induced decoherence but also enabling qubit control through *g*-TMR, both strongly anisotropic in strength with respect to the magnetic field orientation. Furthermore, the hyperfine interaction between the qubit and the ^73^Ge nuclear spin bath is extremely anisotropic and only suppressed when the qubit quantization axis aligns with the quantum-well plane. As a result, the hyperfine interaction is detrimental to qubit coherence for any ${{{\bf{B}}}}\nparallel {x}^{{\prime} }{y}^{{\prime} }$. When the nuclear spin noise can be mitigated, we find qubit coherence to be limited by charge noise with a 1/*f* power spectrum, where the electric field sensitivity originates from *g*-tensor modulation, resulting in a coherence time that is inversely proportional to the qubit energy splitting. We find that qubit coherence can thus be substantially increased by operating in the low-field regime, while maintaining high-fidelity single-qubit control with a gate fidelity well above the fault-tolerant threshold, even at operation temperatures above 1 K. The hyperfine interaction hinders leveraging of the electric field sensitivity sweet spots that would enable a substantial further improvement in qubit coherence, underpinning the need for isotopic purification of the germanium quantum well^44^. This understanding of the dominant decoherence mechanisms and sweet spots for hole spins is key for the future design and operation of large-scale, high-fidelity spin qubit arrays.

## Methods

### Device fabrication

The quantum-dot device is fabricated on a Ge/SiGe heterostructure consisting of a 20-nm-thick quantum well buried 48 nm below the wafer surface, grown in an industrial reduced-pressure chemical vapour deposition reactor^26^. The virtual substrate consists of a strain-relaxed germanium layer on a silicon wafer and multiple layers with increasing silicon content to reach the Si_0.2_Ge_0.8_ stoichiometry used for the quantum-well barriers. Ohmic contacts to the quantum well are defined by the in-diffusion of Pt at a temperature of 300 °C. We note that, in the device used for this work, the platinum silicide did not diffuse in deep enough to reach the quantum well, resulting in a larger contact resistance (at the megaohm level). Electrostatic gates are defined using electron-beam lithography and lift-off of a Ti/Pd alloy (20 nm), separated by thin (7 nm) layers of atomic-layer-deposited SiO_2_.

### Experimental setup

All measurements are performed in a Bluefors LD400 dilution refrigerator with a base temperature of *T* = 10 mK. The sample is mounted on a QDevil QBoard circuit board, and static biases are applied to the gates using a QDevil QDAC digital-to-analogue converter through a twisted-pair wiring loom filtered using a QDevil QFilter low-pass filter at the millikelvin stage of our fridge. In addition, all plunger and barrier gates are connected to coaxial lines through on-PCB bias tees. All coaxial lines are attenuated by 10 dB at the 4K stage and an additional 3 dB at the still. We use Tektronix AWG5204 arbitrary waveform generators (AWGs) to deliver fast voltage excitation pulses to the quantum-dot gates. Furthermore, we use the AWGs to drive the vector input of a Rohde & Schwarz SGS100A source to generate microwave control signals when *f*_Q_ > 500 MHz. For experiments with *f*_Q_ < 500 MHz, we directly synthesize the qubit drive pulses using the AWG. Unfortunately, the coaxial line connected to gate P1 was defective at the time of the experiments. To enable fast pulsing throughout the charge stability diagram of the double quantum dot, we applied pulses to the coaxial line connected to RB1, the reservoir side gate of Q1 (Fig. 1), instead and account for the difference in dot-gate capacitance between P1 and RB1. The independent control over the direct-current voltage on RB1 and P1 still enables a reservoir tunnel rate that is suitable for the experiments to be selected.

The qubits are read out using a charge sensor defined in the lower channel of the four-quantum-dot device. We tune the device to form a single quantum dot underneath the central gate SP, with the tunnel rates being controlled by SB1 and SB2 as defined in Fig. 1. We measure the sensor conductance using a pair of Basel Precision Instruments SP983c transimpedance amplifiers with a gain of 10^6^ and a low-pass output filter with a cut-off frequency of 30 kHz and applying a source–drain bias excitation of *V*_SD_ = 300–800 μV. We directly extract the differential current using a Basel Precision Instruments SP1004 differential amplifier with an additional gain of 10^3^ and record the signal using an Alazar ATS9440 digitizer card.

An external magnetic field is applied through an American Magnetics three-axis magnet with a maximum field of 1/1/6 tesla in the *xyz* direction and a high-stability option on all coils. We note that, owing to an offset *z* = 2.78 cm of the sample with respect to the *xy* coil centres, a correction of –11.2% is applied to *B*_*x*_ and *B*_*y*_ as following from a simulation of the magnet coil fields. As the sample is correctly centred with respect to the *z* solenoid, no off-diagonal components of the applied magnetic field are present (that is, *B*_*x*−coil_∥*x*, *B*_*y*−coil_∥*y* and *B*_*z*−coil_∥*z*). The correctly observed gyromagnetic ratio of the ^73^Ge nuclear spin confirms the accuracy of this correction. Small common rotations of the Q1 and Q2 *g*-tensor rotations may occur due to imperfect planar mounting of the sample. Finally, we note that our magnet coils typically show a few millitesla of hysteresis, which becomes relevant at very low fields. To ensure operation in a hyperfine sweet spot, we sweep *θ*_**B**_ before every measurement in Fig. 6 and locate the sweet plane by minimizing the qubit frequency as a function of *θ*_**B**_.

### Virtual gate matrices

To compensate for the cross-capacitance between the different electrostatic gates and the quantum dots, we define a set of virtual gates^45^:

$$\left(\begin{array}{c}{V}_{{{{\rm{P}}}}1}\\ {V}_{{{{\rm{P}}}}2}\\ {V}_{{{{\rm{P}}}}3}\\ {V}_{{{{\rm{P}}}}4}\\ {V}_{{{{\rm{B}}}}12}\end{array}\right)=\left(\begin{array}{ccccc}1&-0.28&0&0&-1.65\\ -0.18&1&0&0&-1.30\\ 0&-0.11&1&0&0.10\\ -0.11&0&0&1&0.10\\ 0&0&0&0&1\end{array}\right)\left(\begin{array}{c}{V}_{\overline{{{{\rm{P}}}}1}}\\ {V}_{\overline{{{{\rm{P}}}}2}}\\ {V}_{\overline{{{{\rm{SB}}}}2}}\\ {V}_{\overline{{{{\rm{SB}}}}1}}\\ {V}_{\overline{{{{\rm{B}}}}12}}\end{array}\right)$$

with G_*i*_ the real gate voltage and $\overline{{{{\mbox{G}}}}_{i}}$ the virtual gate voltage, which leaves the chemical potential of the other quantum dots unchanged. Furthermore, we define a second pair of axes for detuning *ϵ* and on-site energy *U*, as illustrated in Fig. 1b:

$$\left(\begin{array}{c}{V}_{\overline{{{{\rm{P}}}}1}}\\ {V}_{\overline{{{{\rm{P}}}}2}}\end{array}\right)=\left(\begin{array}{cc}-0.5&0.5\\ 0.5&0.5\end{array}\right)\left(\begin{array}{c}{V}_{\epsilon }\\ {V}_{{{U}}}\end{array}\right)$$

### Pauli spin blockade readout

To overcome rapid spin relaxation as mediated by the spin–orbit interaction^46^, we make use of charge latching, where we tune the tunnel rates between each dot and its respective reservoir to be asymmetric *t*_Q2_ ≪ *t*_Q1_. By pulsing across the extended (1,1)–(0,1) charge-transition line, we can latch the blocking (1,1) states into a (0,1) charge state^8,28^, with a characteristic decay time to the (0,2) ground state governed by *t*_Q2_. Furthermore, the spin–orbit interaction introduces a coupling between the triplet $\left\vert T(1,1)\right\rangle$ and singlet $\left\vert S(0,2)\right\rangle$ states, resulting in the presence of an anticrossing between the $\left\vert \downarrow \downarrow \right\rangle$ and the $\left\vert S(0,2)\right\rangle$ states. As a result, depending on the sweep rate across the interdot transition line, as well as the orientation of the external magnetic field *B*, we observe either parity or single-state readout^8,47^. We typically operate the device in single-state readout by sweeping fast across the anticrossing, unless this was prohibited due to the finite bandwidth of our setup with respect to the different tunnel rates.

Because Pauli spin blockade readout measures the relative spin projection of two qubits, we observe readout to be affected for magnetic field orientations that lead to a large angle between the two qubit quantization axes. We find that readout is completely suppressed when the angle between **f**_Q1_ and **f**_Q2_ equals π/2 (Extended Data Fig. 1f,g).

### Fitting procedure of the *g*-tensor

The *g*-tensor of the device can be described as a rotated diagonal matrix:

1 $${\overleftrightarrow{g}}=R(\phi ,\theta ,\zeta )\,{{{\rm{diag}}}}\,({g}_{{x}^{{\prime} }},{g}_{{y}^{{\prime} }},{g}_{{z}^{{\prime} }}){R}^{-1}(\phi ,\theta ,\zeta )$$

where the Euler angles *ϕ*, *θ* and *ζ* define the successive intrinsic rotations around the *zyz* axes. The rotation matrix *R* is thus defined as:

2 $$R(\phi ,\theta ,\zeta )=\left(\begin{array}{ccc}\cos (\phi )\cos (\theta )\cos (\zeta )-\sin (\phi )\sin (\zeta )&-\sin (\phi )\cos (\zeta )-\cos (\phi )\cos (\theta )\sin (\zeta )&\cos (\phi )\sin (\theta )\\ \sin (\phi )\cos (\theta )\cos (\zeta )+\cos (\phi )\sin (\zeta )&\cos (\phi )\cos (\zeta )-\sin (\phi )\cos (\theta )\sin (\zeta )&\sin (\phi )\sin (\theta )\\ -\sin (\theta )\cos (\zeta )&\sin (\theta )\sin (\zeta )&\cos (\theta )\end{array}\right)$$

The *g*-tensor can thus be reconstructed by measuring the qubit energy splitting *hf*_Q_ for different orientations of the magnetic field **B**. We measure *f*_Q_ for various magnetic field orientations (*θ*_**B**_, *ϕ*_**B**_) and fit the data to:

3 $$h{f}_{{{{\rm{Q}}}}}=\left\vert {\mu }_{{\mathrm{B}}}{\overleftrightarrow{g}}\;{{{\bf{B}}}}\right\vert$$

using ${\overleftrightarrow{g}}$ as defined in equations (1) and (2) and *g*_x′_, *g*_y′_, *g*_z′_, *ϕ*, *θ* and *ζ* as fitting parameters. The data used for the fitting include but are not limited to the data presented in Fig. 2. All magnetic field orientations at which *f*_Q_ is measured are shown in Supplementary Fig. 1c. These field orientations (*θ*_**B**_, *ϕ*_**B**_) are selected to enable a reliable fit of ${\overleftrightarrow{g}}$, with the error on the different parameters indicated in Fig. 2h.

### Fitting procedure of the charge-noise-limited coherence

We measure the qubit coherence by extracting the Hahn echo coherence time, which is insensitive to quasi-static noise and experimental parameters such as the integration time. We measure the normalized charge sensor current as a function of the total free evolution time 2*τ* and observe two different regimes (Extended Data Fig. 2). In the first regime, the echo data follow an exponential decay and we fit the data to $${I}_{{{{\rm{sensor}}}}}=\exp (-{(2\tau /{T}_{2}^{\,{\rm{H}}})}^{\alpha })$$, with the exponent *α* left free as a fitting parameter. However, for magnetic field orientations where the echo decay is dominated by the nuclear spin-induced decoherence ($${{{\bf{B}}}}\nparallel {x}^{{\prime} }{y}^{{\prime} }$$), we extract the envelope coherence $${T}_{2}^{\,{\rm{H}}}$$ by fitting the envelope of the nuclear spin-induced collapse and revival^34^ to $${I}_{{{{\rm{sensor}}}}}=\exp (-{(2\tau /{T}_{2}^{\,{\rm{H}}})}^{\alpha })$$$$/| 1-{a}_{0}\cos (2\uppi {f}_{{{{\rm{Ge}}}}{{\textit{-}}}73}\tau ){| }^{2}$$, with *a*_0_ and *α* free fitting parameters and *f*_Ge-73_ = *γ*_Ge-73_*B*, as discussed further in the main text.

The exponent of the dependence of the Hahn echo coherence time on both (∂*f*_Q_/∂*V*_*i*_)/*f*_Q_ and *f*_Q_ (Fig. 3f,g), is related to the colour of the electric noise spectrum. Assuming charge noise with a power law noise spectrum *S* ∝ *f*^*α*^ acting on a qubit and following the filter formalism from refs. ^15,33^, we find:

4 $${T}_{2}^{{\mathrm{H}}}\propto {\left(\frac{\frac{\partial {f}_{{{{\rm{Q}}}}}}{\partial {V}_{i}}}{{f}_{{{{\rm{Q}}}}}}({\theta }_{{{{\bf{B}}}}},{\phi }_{{{{\bf{B}}}}}) {f}_{{{{\rm{Q}}}}}(B)\right)}^{\frac{2}{\alpha -1}}$$

Therefore, both the dependence of ${T}_{2}^{\,{\mathrm{H}}}$ on the qubit frequency (by varying *B*; Fig. 3f) and on the electric field sensitivity (by varying *θ*_**B**_ and *ϕ*_**B**_; Fig. 3g) should obey a power law with the exponent $\beta =\frac{2}{\alpha -1}$. From this we can derive the noise exponent $\alpha =\frac{2}{\beta }+1$, such that *α* = −1 if *β* = −1.

To obtain the expected charge-noise-limited ${T}_{2}^{\,{\mathrm{H}}}$ at *f*_Q2_ = 1 GHz, we fit a power law ${T}_{2}^{\,{\mathrm{H}}}={T}_{2}^{\,{\mathrm{H}}}[1\,\,{{\mbox{GHz}}}\,]\times 1\,\,{{\mbox{GHz}}}\,/{f}_{{{{\rm{Q}}}}2}$ to the data in Fig. 3f where *B* > *B*_hyperfine_ (opaque markers). Here, *B*_hyperfine_ indicates the magnetic field strength below which the finite spread of the nuclear spin precession frequencies limits qubit coherence^34^.

Because of the limited maximum field strength we can apply along the *x* and *y* axis *B*_max,x_ = *B*_max,y_ = 1 T, the electric field sensitivity for the pink data point is obtained at a lower qubit frequency *f*_Q2_ = 785 MHz and is extrapolated to *f*_Q2_ = 1.36 GHz.

### Fitting procedure of the hyperfine noise

We follow the method presented in refs. ^33,41,48^ and assume a noise spectrum acting on the qubit consisting of a 1/*f* noise spectrum caused by a large number of charge fluctuators and a Gaussian line caused by the hyperfine interaction with the precession of the ^73^Ge nuclear spins:

5 $$\begin{array}{rcl}{S}_{{f}_{Q}}(\;f,{{{\bf{B}}}})&=&{S}_{{{{\mathrm{HF}}}}}(\;f,{{{\bf{B}}}})+{S}_{{\mathrm{E}}}(\;f,{{{\bf{B}}}})\\ {S}_{{{{\mathrm{HF}}}}}(\;f,{{{\bf{B}}}})&=&{S}_{0,{{{\mathrm{HF}}}}}({{{\bf{B}}}})\exp \left(-\displaystyle\frac{f-{\gamma }_{{{{\rm{Ge}}}}{{\textit{-}}}73}B}{2{\sigma }_{{{{\rm{Ge}}}}{{\textit{-}}}73}^{2}}\right)\\ {S}_{{\mathrm{E}}}(\;f,{{{\bf{B}}}})&=&\displaystyle\frac{{S}_{0,{\mathrm{E}}}}{f}({{{\bf{B}}}})=\frac{{S}_{0,{\mathrm{V}}}{\left(\displaystyle\frac{\partial {f}_{{{{\rm{Q}}}}}}{\partial {V}_{{{{\rm{P}}}}2}}({{{\bf{B}}}})\right)}^{2}}{f}\end{array}$$

Here, *S*_0,HF_(**B**) defines the effective strength of the nuclear spin noise acting on the qubit, which can be related to the hyperfine coupling constants as detailed below. Furthermore, *γ*_Ge-73_ = 1.48 MHz T^−1^ is the ^73^Ge gyromagnetic ratio and *σ*_Ge-73_ represents the finite spread of the ^73^Ge precession frequencies. The charge noise acting on the qubit is most probably originating from charge traps in the interfaces and oxides directly above the qubit, so we model its coupling as coming from the qubit plunger gate, in agreement with what we find in Fig. 3. *S*_0,V_ is the effective voltage noise power spectral density and $\frac{\partial {f}_{{{{\rm{Q}}}}2}}{\partial {V}_{{{{\rm{P}}}}2}}({{{\bf{B}}}})$ is the sensitivity of the qubit frequency to electric potential fluctuations from the plunger gate P2. The qubit will undergo dephasing as a result of the energy splitting noise, which will lead to a decay as defined by:

6 $$P(\tau )\propto \exp \left(-{{{\mathcal{X}}}}(\tau )\right)$$

with *P* the measured spin-up probability and

7 $${{{\mathcal{X}}}}(\tau )=\int\nolimits_{0}^{\infty }{S}_{{\omega }_{Q}}(\omega )\frac{{F}_{N}(\omega ,\tau )}{\uppi {\omega }^{2}}{\mathrm{d}}\omega$$

with *S*_*ωQ*_ the qubit detuning noise and *ω* the angular frequency. The unitless filter function *F*_*N*_ for the CPMG experiment is defined as follows^33^:

8 $${F}_{N}(\omega\tau)=\left\{\begin{array}{ll}8\,{\sin }^{4}\left(\omega\tau /4\right)\displaystyle\frac{{\sin }^{2}\left(N\omega \tau /2\right)}{{\cos }^{2}\left(\omega\tau /2\right)},\quad &N\,\,{{\mbox{is even}}}\,\\ 8\,{\sin }^{4}\left(\omega \tau /4\right)\displaystyle\frac{{\cos }^{2}\left(N\omega\tau /2\right)}{{\cos }^{2}\left(\omega\tau/2\right)},\quad &N\,\,{{\mbox{is odd}}}\,\end{array}\right.$$

As both the strength of the nuclear spin noise and charge noise are expected to depend on **B**, we fit the data for each *θ*_**B**_ independently, fixing *γ*_Ge-73_ = 1.48 MHz T^−1^ and keeping *σ*_Ge-73_, *S*_0,V_ and *S*_0,HF_ as fit parameters.

We note that we find *σ*_Ge-73_ to be independent of *θ*_**B**_ within the experimental range, with an average ${\overline{\sigma }}_{{{{\rm{Ge}}}}{{\textit{-}}}73}$ = 9 kHz (Extended Data Fig. 5e,f). This line width is several orders of magnitude larger than expected for a single ^73^Ge spin^49^, but it is in good agreement with values previously observed in germanium^39^. The finite width of the hyperfine line is mostly reflected in the loss of the coherence for low magnetic fields, when *f*_Ge-73_ ≈ *σ*_Ge-73_. This can be observed in the data presented in Fig. 3f, as well as when performing the CPMG experiment as a function of the magnetic field strength (Extended Data Fig. 6). However, we observe this line width to be dependent on the azimuth orientation of the external magnetic field *ϕ*_**B**_ (Extended Data Fig. 5), potentially indicative of a quadrupolar origin, which would depend on strain and electric fields and thus be dependent on the magnetic field orientation.

Increasing the number of refocusing pulses also sharpens the effective bandpass filter of the CPMG sequence^33,50^, thus enhancing the sensitivity of the qubit to the nuclear spin precession. As a result, a higher accuracy of *θ*_**B**_ is required to align exactly to the hyperfine sweet spot and avoid loss of coherence due to hyperfine interaction with the ^73^Ge nuclear spins. This is illustrated in Supplementary Fig. 2, where we measure the CPMG decay as a function of the number of refocusing pulses *N*.

### Estimation of the hyperfine coupling constant

The reconstruction of the hyperfine noise spectrum enables estimation of the hyperfine coupling constants for a heavy hole in germanium. From the fit to the data in Fig. 5, we have *S*_0,HF_ = 2.52(4) kHz^2^ Hz^−1^ for an out-of-plane field and *σ*_Ge-73_ = 9.9(11) kHz. This equates to an integrated detuning noise of:

9 $${\sigma }_{f}=\sqrt{\sqrt{2\uppi }{S}_{0,{{{\rm{HF}}}}}{\sigma }_{{{{\rm{Ge}}}}{{\textit{-}}}73}}=250\,{{\mbox{kHz}}}$$

Assuming a Gaussian noise distribution, this corresponds to an expected phase coherence time^51^ of ${T}_{2}^{\,{*}}=1/(\uppi \sqrt{2}{\sigma }_{f})=900\,{{\mbox{ns}}}$. We can estimate the out-of-plane hyperfine coupling *A*_∥_ using equation (2.65) from ref. ^52^:

10 $${h}^{2}{{\sigma }_{f}}^{2}\approx \frac{1}{4N}{g}_{{{{\rm{Ge}}}}{{\textit{-}}}73}I(I+1){A_{\parallel }}^{2}$$

such that:

11 $${A}_{\parallel}\approx \sqrt{\frac{4N}{{g}_{{{{\rm{Ge}}}}{{\textit{-}}}73}I(I+1)}}h{\sigma }_{f}$$

with *g*_Ge-73_ = 0.0776 the natural abundance of the ^73^Ge isotope, *I* = 9/2 the ^73^Ge nuclear spin and *N* the number of nuclei the quantum dot wavefunction overlaps. To estimate *N*, we consider a cylindrical quantum dot, such that *N* = π*r*^2^*w*/*v*_0_, where *r* is the radius, *w* is the height of the dot and *v*_0_ = 2.3 × 10^−29^ m^3^, the atomic volume of germanium. We can estimate *r* from the single-particle-level splitting Δ*E* ≈ 1.2 meV, as can be obtained from the extent of the Pauli spin blockade readout window, and find *r* ≈ 35 nm. This is in good agreement with *r* ≈ 50 nm as expected from the charging energy *E*_C_ ≈ 2.8 meV and the capacitance of a disc: *r* = *e*^2^/(8*ϵ*_*r*_*E*_*C*_), with *ϵ*_*r*_ the relative dielectric constant and *e* the elementary charge. Assuming *r* = 35 nm and *w* = 10 nm (half of the quantum-well width), we then find *N* ≈ 1.7 × 10^6^. Using equation (11), we estimate the hyperfine coupling constant to be ∣*A*_∥_∣ ≈ 1.9 μeV, which is in good agreement with the theoretical prediction of *A*_∥_ = −1.1 μeV from ref. ^40^. Similarly, from the extracted *S*_0,HF_ for an in-plane **B**, we estimate an upper bound for the in-plane hyperfine coupling constant *A*_⊥_of <0.1 μeV, which is compatible with the predicted *A*_⊥_ = 0.02 μeV.

### Randomized benchmarking

To extract the single-qubit gate fidelity, we perform randomized benchmarking of the Clifford gate set presented in Supplementary Table 2. For every randomization, we measure both the projection to $\left\vert \uparrow \right\rangle$ and $\left\vert \downarrow \right\rangle$ and fit the difference to avoid inaccuracies due to the offset of the charge sensor current. The measured current is normalized to the signal obtained from a separate measurement of our $\left\vert \uparrow \right\rangle$ and $\left\vert \downarrow \right\rangle$ states. We fit the data to *P* = *a* exp(−2(1 − *F*_C_)*N*_C_), where *F*_C_ is the Clifford gate fidelity and *N*_C_ is the number of applied Clifford gates. The parameter *a* is an additional scaling parameter we include to account for the reduced visibility we observe when applying a large number of radiofrequency pulses. Fixing *a* = 1 does not significantly alter the fit, as shown by the dotted line in Fig. 6. In fact, we find *F*_g_ = 99.94% for *T* = 20 mK and *F*_g_ = 99.7% for *T* = 1.1 K when fixing *a* = 1. The primitive gate fidelity *F*_g_ can be calculated by accounting for the average number of physical gates (*I*, *X*/2, *X*) per Clifford: 0.875 for this gate set.

### Extraction of the *g*-tensor sensitivity

We measure the modulation of the qubit energy splitting *δf*_Q_ as the result of a small voltage pulse *δV* applied to one of the quantum-dot gates. The voltage pulse will temporarily shift the qubit resonance frequency, thus inducing an effective phase gate, controlled by the length of the pulse *t*_*Z*_. By incorporating this phase gate within the free evolution of a Hahn echo experiment, we can observe the phase oscillations as a function of *t*_*Z*_, as shown in Fig. 3c. From the frequency of these oscillations, we obtain ∣*δf*_Q_∣. We confirm that for a small *δV*, ∣*δf*_Q_∣ is linear in *δV*, enabling us to extract the sensitivity ∣∂*f*_Q_/∂*V*_*i*_∣ from a single data point of *δV* (Supplementary Fig. 3). To exclude effects caused by the exchange interaction *J* between the qubits, we tune *J* < 1 MHz using the interdot barrier B12. Furthermore, we tune the device to the point of symmetric exchange in the (1,1) region^53,54^ and apply symmetric pulses in the first and second free evolution period of the Hahn sequence, echoing out effects caused by changes of the double dot detuning. To extract the sign of ∂*f*_Q_/∂*V*_*i*_, we measure the qubit resonance frequency for three different gate voltage settings (Fig. 3c) for a few selected magnetic field orientations.

Given a g-tensor ${\overleftrightarrow{g}}$ and a g-tensor sensitivity $\partial\;{\overleftrightarrow{g}}/\partial {V}_{i}$, ∂*f*_Q_/∂*V*_*i*_ only depends on the magnetic field direction **b** and on *f*_Q_ ∝ *B*:

12 $$\frac{\delta {f}_{{{{\rm{Q}}}}}}{\delta V}({\theta }_{{{{\bf{B}}}}},{\phi }_{{{{\bf{B}}}}},B)=\frac{\left(\partial\;{\overleftrightarrow{g}}/\partial {V}_{i} {{{\bf{b}}}}\right)\cdot \left(\overleftrightarrow{g}\;{{{\bf{b}}}}\right)}{{\left({\overleftrightarrow{g}}\;{{{\bf{b}}}}\right)}^{2}}{f}_{{{{\rm{Q}}}}}(B)$$

We extract $\partial\;{\overleftrightarrow{g}}/\partial{V}_{i}$ by fitting equation (12) to the data presented in Fig. 4, using ${\overleftrightarrow{g}}$ as extracted previously and displayed in Fig. 2h. We then calculate the expected *g*-TMR-mediated Rabi frequency using

13 $$\frac{\delta {f}_{{{{\rm{Rabi}}}}}}{\delta V}({\theta }_{{{{\bf{B}}}}},{\phi }_{{{{\bf{B}}}}},B)=\mu \frac{\left\vert \left(\partial\;{\overleftrightarrow{g}}/\partial {V}_{i} {{{\bf{b}}}}\right)\times \left(\;{\overleftrightarrow{g}}\;{{{\bf{b}}}}\right)\right\vert }{2|\;{\overleftrightarrow{g}}\;{{{\bf{b}}}}{| }^{2}}{f}_{{{{\rm{Q}}}}}(B)$$

where *f*_Rabi_ is the Rabi frequency and *μ* the signal attenuation for a microwave signal at a frequency of *f*_Q_.

We fit the data to equation (13), with *μ* as the only fit parameter. We find an additional line attenuation of *μ*_P2_ = 0.47, *μ*_B2_ = 0.47 and *μ*_B12_ = 0.50 at this frequency. These values are in good agreement with the attenuation of our experimental setup at *f* = 225 MHz as extracted from the broadening of the charge sensor Coulomb peak (*μ* = 0.40–0.47) (Supplementary Fig. 4).

## Online content

Any methods, additional references, Nature Portfolio reporting summaries, source data, extended data, supplementary information, acknowledgements, peer review information; details of author contributions and competing interests; and statements of data and code availability are available at 10.1038/s41563-024-01857-5.

### Supplementary information

Supplementary Information Supplementary Tables 1 and 2 and Figs. 1–4.

## Data Availability

All data underlying this study are available at Zenodo at 10.5281/zenodo.7986574 (ref. ^55^).

## Extended data

**Extended Data Table 1:** Description of ${{\partial\;}}{{\overleftrightarrow{\boldsymbol{g}}}}/{{\partial}} {\boldsymbol{V}}_{\boldsymbol{i}}$ of Q2 for a potential applied to gates P2, B2, and B12

$\partial\;{\overleftrightarrow{g}}/\partial {V}_{i}$	P2	B2	B12
∂*ϕ*/∂*V*_*i*_ (mrad ⋅ mV^−1^)	-0.11(2)	1.3(2)	-3.1(1)
∂*θ*/∂*V*_*i*_ (mrad ⋅ mV^−1^)	0.0008(5)	0.005(4)	0.028(3)
∂*ζ*/∂*V*_*i*_ (mrad ⋅ mV^−1^)	0.04(2)	-2.3(1)	2.2(1)
$\partial {g}_{{x}^{{\prime} }}/\partial {V}_{i}$ (mV^−1^)	0.000181(9)	-0.00028(7)	-0.00073(6)
$\partial {g}_{{y}^{{\prime} }}/\partial {V}_{i}$ (mV^−1^)	0.000507(3)	0.00037(2)	-0.00146(2)
$\partial {g}_{{z}^{{\prime} }}/\partial {V}_{i}$ (mV^−1^)	0.0045(1)	-0.0001(8)	-0.0071(7)

Overview of the parameters describing the voltage-induced deformation of ${\overleftrightarrow{g}}$. Three Euler angles ∂*ζ*/∂*V*_*i*_, *θ*/∂*V*_*i*_, and ∂*ϕ*/∂*V*_*i*_ describing the rotation, as well as changes to the principle *g*-factors $\partial {g}_{{x}^{{\prime} }}/\partial {V}_{i},\partial {g}_{{y}^{{\prime} }}/\partial {V}_{i}$, and $\partial {g}_{{z}^{{\prime} }}/\partial {V}_{i}$, for Q2.
