# Supplementary material for: Sweet-spot operation of a germanium hole spin qubit with highly anisotropic noise sensitivity
Source: Nat Mater. 2024 May 17;23(7):920–7. doi: 10.1038/s41563-024-01857-5 (PMC11230914; doi:10.1038/s41563-024-01857-5)
Supplement: Supplementary file 1 — Supplementary Tables 1 and 2 and Figs. 1–4. [file 41563_2024_1857_MOESM1_ESM.pdf]

nature materials

Article

https://doi.org/10.1038/s41563-024-01857-5

Sweet-spot operation of a germanium hole spin qubit with highly anisotropic noise sensitivity

In the format provided by the authors and unedited

CONTENTS

Supplementary Table I 2
Supplementary Table II 2
Supplementary Figure 1 3
Supplementary Figure 2 3
Supplementary Figure 3 4
Supplementary Figure 4 4

Data set	ϕ_B (deg.)	θ_B (deg.)
●	0.0	90.03
▼	0.0	85.50
▲	0.0	95.63
◀	-105.0	95.63
▶	-105.0	90.00
✚	-105.0	89.91
✖	0.0	91.13
◆	0.0	91.08

Supplementary Table I. **Magnetic field parameters for data in Fig. 3 of the main text.** Magnetic field elevation θ_B and azimuth ϕ_B for the different data sets in Fig. 3 of the main text, as indicated by the coloured markers.

Clifford gates
I
-Z, X
-Z, X/2
-Z, X/2, -Z
-Z, X/2, -Z/2
-Z, X/2, Z/2
-Z/2
-Z/2, X/2
-Z/2, X/2, -Z
-Z/2, X/2, -Z/2
-Z/2, X/2, Z/2
X
X, Z/2
X/2
X/2, -Z
X/2, -Z/2
X/2, Z/2
Z
Z/2
Z/2, X
Z/2, X/2
Z/2, X/2, -Z
Z/2, X/2, -Z/2
Z/2, X/2, Z/2

Supplementary Table II. **Clifford set used for randomized benchmarking.** The Clifford set used for the randomized benchmarking consists out of $X/2$ and X pulses, and virtual Z gates. The average number of physical gates per Clifford equals 0.875.

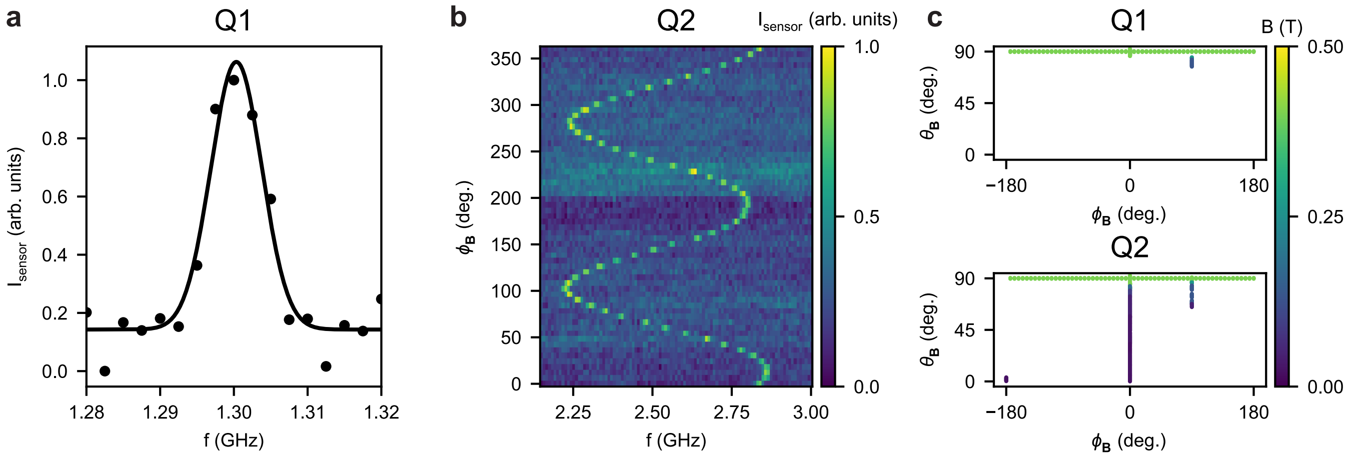

Supplementary Figure 1. **Exemplary EDSR resonance traces used to fit the qubit g-tensors.** **a**, Normalized charge sensor current, as a function of the microwave frequency f, showing spin resonance for Q1. We apply a frequency chirp to assure full spin inversion. **b**, Normalized charge sensor current, as a function of the microwave frequency f and magnetic field azimuth $\phi_{\mathbf{B}}$. **c**, Illustration of all magnetic field orientations at which g^* was measured to fit $\overleftrightarrow{g}$, for Q1 (top) and Q2 (bottom). Each of the 402 (935) markers corresponds to a single resonance measurement for Q1 (Q2) as in panel **a**.

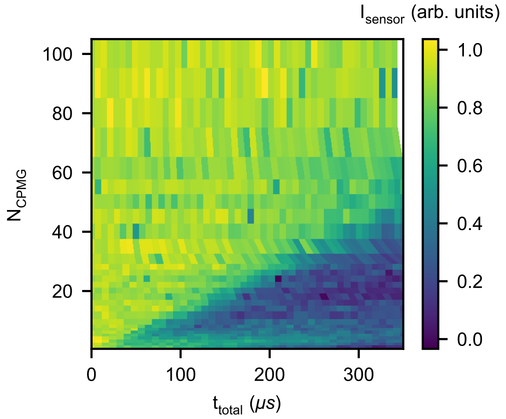

Supplementary Figure 2. **CPMG dynamical decoupling as a function of the number of decoupling pulses.** Charge sensor signal as a function of the total evolution time and number of decoupling pulses of a CPMG sequence. The magnetic field is aligned to the hyperfine sweet spot. For low N_{CPMG} we observe an expected Gaussian decay due to charge noise, but for increasing N the sensitivity to the nuclear noise is enhanced and we observe a sharp coherence collapse caused by the hyperfine interaction.

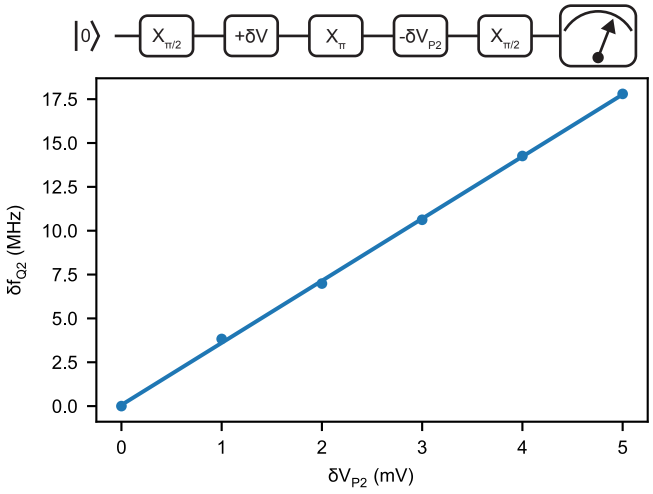

Supplementary Figure 3. **Linearity of the qubit frequency shift.** We apply a Hahn echo sequence to the qubit, where a positive (negative) voltage pulse $\delta V_{\rm P2}$ is applied during the first (second) free evolution, as illustrated in the schematic. As we vary the depth of the voltage pulse, we observe a linear shift of the qubit frequency, which allows us to extract $\partial f_{\rm Q2}/\partial V_{\rm P2}$ by evaluating the frequency shift for a single point of $\delta V_{\rm P2}$.

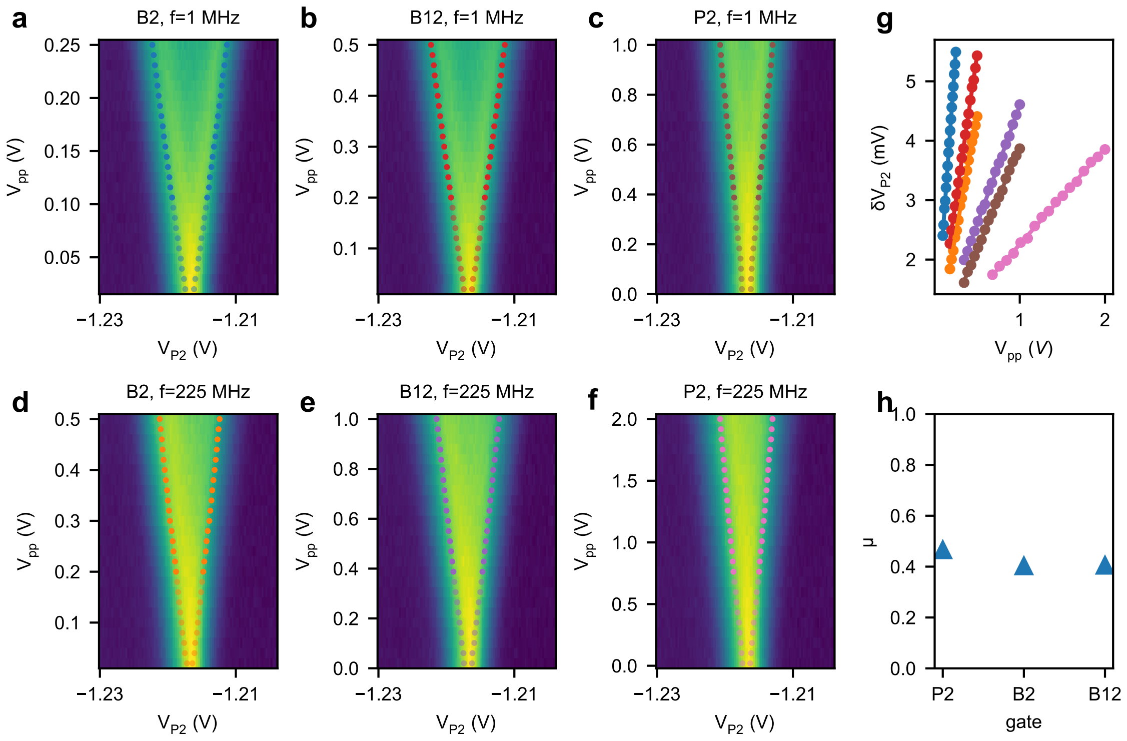

Supplementary Figure 4. **Attenuation of the microwave pulses applied to different gates.** **a-c**, Charge sensor Coulomb peak as a result of a continuous wave (CW) microwave excitation with a frequency of $f_{\rm CW} = 250$ MHz and a peak-to-peak amplitude of $V_{\rm pp}$, applied to gate B2 (**a**), B12 (**b**), and P2 (**c**). Solid markers indicate the regime where the splitting $\delta V_{\rm pp} > \sigma$ with σ the peak width. **d-f**, Charge sensor Coulomb peak as a result of a continuous wave microwave excitation with a frequency of $f_{\rm CW} = 1$ MHz and a peak-to-peak amplitude of $V_{\rm pp}$, applied to gate B2 (**a**), B12 (**b**), and P2 (**c**). Solid markers indicate the regime where the splitting $\delta V_{\rm pp} > \sigma$ with σ the Coulomb peak width. **g** Splitting of the Coulomb peak $\delta V_{\rm pp}$, as extracted by fitting each line to the expected shape of a sine wave broadened Lorentzian, obtained by analytically evaluating the convolution between a Lorentzian and the probability distribution of a sine. We extract the net attenuation factor $\alpha_{f_{\rm CW},i}$ at frequency $f_{\rm CW}$ for gate i, by fitting the data indicated by the solid markers in panels **a-f** to $\delta V_{\rm P2} = \alpha_{f_{\rm CW},i} V_{\rm pp}$. Colour of the markers correspond to the coloured markers as used in panels **a-f**. **h** The ratio between the net attenuation at high and low frequency $\mu_i = \alpha_{250\ {\rm MHz},i}/\alpha_{1\ {\rm MHz},i}$ for the three different gates i. We find attenuation ratios of $\mu_{\rm P2} = 0.47$, $\mu_{\rm B12} = 0.41$, and $\mu_{\rm B2} = 0.40$.
